# Prevalence of and factors associated with low back pain among construction workers in Mogadishu, Somalia

**DOI:** 10.3389/fpubh.2026.1978211

**Published:** 2026-10-22

**Authors:** Abdikani Yasin Haji Ali, Kellen J. Karimi, Marshal M. Mweu

**Affiliations:** Department of Public and Global Health, Faculty of Health Sciences, University of Nairobi, Nairobi, Kenya

**Keywords:** prevalence, associated factors, construction workers, low back pain, occupational health, Somalia

## Abstract

**Background:**

Low back pain (LBP) is a leading cause of disability, particularly in labor-intensive industries. While biomechanical hazards are well-documented in developed nations, epidemiological data from unregulated construction sectors in post-conflict regions remains extremely scarce. This study aimed to estimate the prevalence and identify factors associated with LBP among manual construction workers in Mogadishu, Somalia.

**Methods:**

An analytic cross-sectional study was conducted among 191 manual workers recruited via stratified random sampling from five major construction companies. Data were collected using a culturally-adapted Nordic Musculoskeletal Questionnaire to assess socio-demographic, lifestyle, and occupational exposures. We utilized a multivariable mixed-effects logistic regression model, accounting for clustering by company, to identify independent associated factors. Results are reported as adjusted odds ratios (AOR) with 95% confidence intervals (CI).

**Results:**

The workforce was notably young (median age = 26 years), registering an LBP period prevalence of 51.9% (95% CI: 43.5–60.3%). In the adjusted analysis, income status was the strongest predictor, with workers living below extreme poverty facing elevated odds of LBP (AOR = 7.60; 95% CI: 2.48–23.28). Other factors significantly associated included possessing over six years of work experience (AOR = 4.85; 95% CI: 1.56–15.11), being underweight (AOR = 5.22; 95% CI: 1.24–21.94), tobacco use (AOR = 5.70; 95% CI: 1.94–16.78), and inadequate sleep duration of fewer than six hours (AOR = 4.11; 95% CI: 1.28–13.24). Biomechanically, frequency of heavy lifting (AOR = 4.29; 95% CI: 1.26–14.56) and bending (AOR = 5.35; 95% CI: 1.33–21.45) were strongly associated with reporting LBP.

**Conclusion:**

LBP is highly prevalent among construction workers in Mogadishu. The factors associated with LBP were extreme poverty, inadequate physiological tissue recovery, and severe biomechanical overload. Mitigating this crisis requires site-level ergonomic interventions, such as team-lifting protocols, alongside overarching policies that establish baseline wages to address foundational socioeconomic hazards.

## 1 Introduction

Construction work is one of the most physically demanding and dangerous jobs in the world (1). Laborers face extreme physical stress daily from heavy lifting, awkward body positions, and repetitive bending (2, 3). These strenuous movements place massive pressure on the lower spine and frequently lead to severe musculoskeletal disorders (4, 5). Among these injuries, low back pain (LBP) is the leading cause of disability globally and affects an estimated 1.71 billion people (6).

Chronic back pain ruins a person’s mobility, forces many workers into early retirement, and drastically lowers their quality of life (7). In the construction sector, the number of laborers suffering from this condition is remarkably high (8). Studies show that LBP affects 52% of construction workers in Nepal (9). Similar research in Sweden reports a prevalence of 53.2% (10).

Studies in Saudi Arabia show a 50.0% prevalence rate among manual builders (11). Workers in Japan experience rates around 29.3% (12). These figures underscore a high injury burden that aligns with reports among manual construction laborers in other developing and regional contexts (13, 14). These are well documented in wealthy countries, but data from developing regions remains incredibly scarce (14). Countries in East Africa carry a massive share of the global disability burden caused by poverty and lack of healthcare resources (15). Somalia is currently experiencing a major economic recovery and a rapid construction boom (16). Yet, the country lacks documented occupational health data regarding the workers building its cities. In Mogadishu, construction companies rarely use mechanized lifting equipment and rely almost entirely on informal manual labor.

Research indicates that lifestyle factors like smoking and alcohol consumption significantly increase the risk of LBP (17, 18). Heavy smoking restricts blood flow and prevents spinal tissues from healing properly (10). Workers also suffer from chronic lack of sleep, poor diets, and high mental stress (19, 20). Psychosocial issues such as job insecurity and high work demands take a heavy physical toll on the body (21). Workers who feel overwhelmed by their tasks are far more likely to develop severe musculoskeletal disorders (22). Furthermore, prolonged exposure to whole body vibration from heavy machinery directly damages the lumbar spine over time (23).

These harsh living and working conditions make it nearly impossible for their bodies to recover from the structural damage they take on the job (9). Solving this hidden health crisis requires accurate local data to create realistic safety rules. Therefore, the objectives of this study were to estimate the prevalence of LBP among manual construction workers in Mogadishu, Somalia and to identify the demographic, lifestyle, and physical factors associated with this condition. Identifying these associated factors gives site managers and policymakers the evidence needed to protect their workers.

## 2 Methods

### 2.1 Study design and setting

An analytical cross-sectional study design was employed to estimate the prevalence and investigate the factors associated with (LBP) among manual construction workers in Mogadishu, Somalia, from January 2025 to December 2025. The rapid urbanization in the Banadir region relies heavily on unregulated manual labor. The study was conducted in five major construction companies (Buruuj, Docol, Al Bina, Eameco, and Nawil) in this region- selected on the basis of their operational scale. The study was reported as per the STROBE guidelines for reporting observational studies (24).

### 2.2 Study population, sampling and eligibility participants

The study population comprised active manual construction workers aged 18 years and older who had at least six months of industry experience. No upper age limit was imposed so as to capture all actively employed laborers appearing on company attendance rosters, yielding an observed participant age range of 18 to 66 years. We excluded workers with a history of traumatic spinal cord injuries or deformities. The required sample size was calculated using the Kelsey formula for cross-sectional studies (25).


$$\begin{array}{l} n_{1}=\frac{{\left(Z_{\alpha }-Z_{\beta }\right)}^{2}\bar{\mathrm{pq}}(r+1)}{r{\left(p_{1}-p_{2}\right)}^{2}}\\ \bar{q}=1-\bar{p}\;\;\;\;\;\;\;\;\;\;\;\;\;\;n_{2}=rn_{1} \end{array}$$

$$p_{1}=\frac{p_{2}\text{OR}}{1+p_{2}(\text{OR}-1)}$$

$$\bar{p}=\frac{p_{1}+rp_{2}}{\left(r+1\right)}$$

Where:

*n*₁ is the number of unexposed participants and *n*₂ is the number of exposed participants; *p*₁ is the proportion of unexposed individuals (workers not engaged in heavy lifting) with LBP, estimated at 31% based on Burdorf et al. (23); *p*₂ is the proportion of exposed individuals (workers engaged in heavy lifting) with LBP, estimated at 59% (23). Z*α*/2 (1.96) and Z1–*β* (0.84) specify the desired two-tailed 95% confidence level and 80% statistical power, respectively. The odds ratio (OR) for the effect of the primary exposure (heavy lifting) on LBP was hypothesized to be 3.20 (23). The ratio (*r*) of unexposed to exposed individuals was set at 1.

Based on these parameters, Kelsey’s formula yielded an initial baseline sample size of 100 participants. To preserve statistical power when adjusting for multivariable confounding, this baseline requirement was expanded following the methodological guidelines of Dohoo et al. (26), which recommend increasing the sample size by approximately 15% per additional covariate anticipated in multivariable modeling. Accounting for five key additional occupational, socioeconomic, and lifestyle exposures (forward trunk flexion, whole-body vibration, job demands, income status, and cumulative work experience), the adjusted target sample size was calculated as 201 participants. To recruit the participants, we employed stratified random sampling. Each of the five selected construction companies was treated as a distinct stratum, and the 201 target participants were proportionally allocated based on the estimated workforce each company. Subsequently, using daily attendance rosters provided by site supervisors as sampling frames, simple random sampling was utilized to select individual participants within each company (Figure 1).

**Figure 1:**
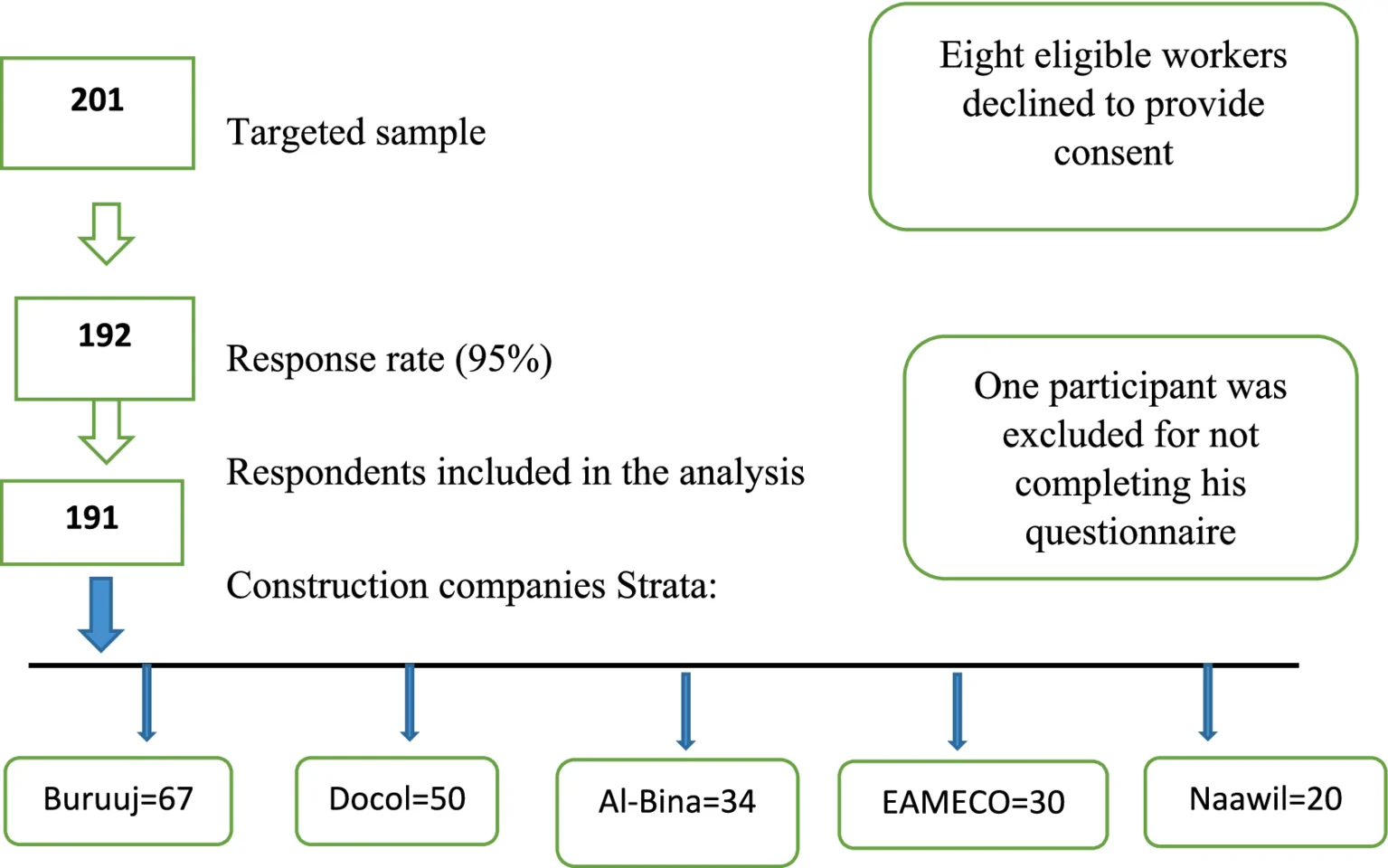
Flow chart of the stratified random sampling strategy.

### 2.3 Outcome definition

An LBP case was strictly defined as a worker reporting ache, pain, or discomfort localized between the lower margin of the twelfth ribs and the lower gluteal folds. The pain had to last for at least 24 h within the preceding 12 months.

### 2.4 Data collection

We utilized a self-administered questionnaire adapted from the standardized Nordic Musculoskeletal Questionnaire (27). The tool was translated into Somali and piloted in a 10% sample to ensure complete comprehension among the laborers. The questionnaire captured socio-demographic data, lifestyle habits, and specific occupational exposures. We measured participant height and weight following the National Health and Nutrition Examination Survey guidelines to calculate body mass index (28) (Table 1).

**Table 1:** Predictor variables and their measurements.

Variables	Measurement of predictor variables
Age (continuous)	Captured in years as reported by each construction worker.
Marital status (categorical)	Classified into single, married, divorced, or widowed.
Income status (continuous)	Monthly income of the worker in Us dollars.
Smoking status (categorical)	Participants were classified as smokers (yes) or non-smokers (no).
Alcohol consumption (categorical)	Reported as yes (consumer) or no (non-consumer).
Sleep pattern (categorical)	Duration of sleep as reported by the participants.
Body mass index (continuous/categorical)	Calculated from measured height and weight (kg/m^2^) and categorized according to WHO standards (58): Underweight (<18.5 kg/m^2^), Normal weight (18.5–24.9 kg/m^2^), and Overweight (≥25.0 kg/m^2^).
Psychosocial distress (binary composite)	A composite score capturing cumulative workplace mental strain, calculated by summing three occupational stress items: job insecurity, perceived job stress, and job demands (observed score range: 2–8). Operationalized as a binary variable based on the sample distribution (upper-tertile split): ‘Low/Moderate Distress’ (≤ 5; 62.3% of workers, *n* = 119) and ‘High Distress’ (> 5; upper 37.7% of workers, *n* = 72).
Job type (nominal)	Worker’s designated role in the construction site.
Years of work (categorical)	Number of years in the construction field.
Daily work hours (categorical)	Typical number of hours worked per day.
Frequency of heavy lifting (categorical)	Frequency of heavy lifting, categorized as never/occasionally (<2 h per day) and frequently (≥2 h per day).
Bending (categorical)	Frequency of bending during work tasks, categorized as rarely (<1), occasionally (1–3 h/day), or frequently (>3 h/day).
Vibration exposure (categorical)	Frequency of exposure to equipment vibration, categorized as never, Rarely (< 1 h/day), or frequently (≥1 h/day).

### 2.5 Ethical considerations

The study protocol was reviewed and approved by the Kenyatta National Hospital and University of Nairobi joint Ethics and Research Committee (Approval Ref: KNH-ERC/A/462) in Kenya, as well as the Somali National Institute of Health Institutional Review Board (Approval Ref: NIH/IRB/103/NOV/2025) in Somalia. All participants provided written informed consent prior to participation, and data were anonymized to maintain strict confidentiality.

### 2.6 Minimization of bias

Because cross-sectional designs are inherently vulnerable to epidemiological biases, we implemented strict methodological controls. To mitigate reverse causality where workers might alter their physical or lifestyle habits after experiencing pain our questionnaire specifically anchored exposure variables to the period before symptom onset. Furthermore, to minimize measurement and interviewer bias, we utilized a standardized, translated Somali version of the Nordic Musculoskeletal Questionnaire and rigorously trained all data collectors on uniform administration techniques. To minimize selection bias associated with non-response, we engaged workers directly at the construction sites, resulting in a high response rate.

### 2.7 Statistical analysis

Data were analyzed using Stata version 17.0. Descriptive statistics comprised frequencies and percentages for categorical variables, while continuous variables were summarized using means with standard deviations for normally distributed data, and medians with interquartile ranges (IQR, 25th–75th percentiles) and absolute ranges (minimum–maximum) for skewed variables such as monthly income. To account for potential correlation among laborers sampled from the same construction enterprise, mixed-effects multivariable logistic regression was fitted with Construction Company specified as a random intercept. Given the recruitment structure across five distinct enterprises, this specification was implemented to avoid violating the assumption of independent observations and to produce appropriately adjusted standard errors. The intraclass correlation coefficient was calculated using the latent variable threshold approach to estimate the proportion of variance attributable to workplace-level clustering. At the univariable analysis stage, the effect of each predictor on LBP, was evaluated at a liberal threshold of *p* ≤ 0.20. In the statistical modeling, body mass index (BMI) and age were operationalized as categorical covariates *a priori* based on established epidemiological literature and biological plausibility, avoiding the restrictive assumption of a strictly linear relationship with low back pain log-odds. Specifically, BMI was classified strictly according to standard World Health Organization (WHO) categories: Underweight (<18.5 kg/m^2), Normal weight (18.5—24.9 kg/m^2), and Overweight (≥ 25.0 kg/m^2), allowing for the evaluation of distinct nutritional risk profiles. Age was partitioned into three distinct occupational life-stage cohorts: 18–28 years (representing young apprentice and entry-level laborers), 29–40 years (representing the core, physically peak manual workforce performing high-intensity site tasks), and ≥ 41 years (representing mature, veteran laborers subject to cumulative occupational wear and the healthy worker survivor effect). Monthly income was categorized into poverty status: (above the poverty line and below poverty line <$3/day). Variables that were found to be significant at the univariable stage were then offered to a multivariable model and retained at *p* < 0.05. The results were reported as adjusted odds Ratios with 95 percent confidence intervals.

To minimize confounding, elimination of non-significant covarietes was only considered when their exclusion from the model did not result in a more than 30% change in the effects of the remaining variables. Two-way interactions were fitted between the remaining variables of the final model and assessed for significance. The intra-class correlation coefficient (ICC) was calculated to determine the proportion of variance at the company level. Of note, the 12-month period prevalence of LBP (along with its confidence interval) was estimated from the intercept of the null model.

## 3 Results

### 3.1 Descriptive characteristics and prevalence

The socio-demographic, lifestyle, and occupational characteristics of the study participants are displayed in Table 2. Of 201 invited workers, 191 completed the survey, yielding a 95.0% response rate. Notably, the median age for the participants was 26 years (Range: 18–66). Regarding socio-demographic characteristics, the majority of the workers were married (61.3%, *n* = 117), and the overall median monthly income across all participants was 300 USD (IQR: 220–350 USD; range: 150–1,000 USD). In terms of occupational background, 66.5% (*n* = 127) of the workforce had less than six years of construction experience. Additionally, nearly half of all respondents (47.6%, *n* = 91) had no formal education. Regarding physical and lifestyle factors, 29.3% (*n* = 56) frequently lifted heavy loads. The estimated 12-month period prevalence of LBP from the null mixed-effects logistic regression model was 51.9% (95% CI: 43.5–60.3%).

**Table 2:** Demographic, lifestyle, and occupational characteristics of manual construction workers in Mogadishu, Somalia (*N* = 191).

Variable	Categories	Frequency n (%)	Median (IQR) [Range]
Age (years)	—	—	26 (18–66)
Education level	Any formal education	100 (52.4)	—
	No formal education	91 (47.6)	—
Marital status	Unmarried	74 (38.7)	—
	Married	117 (61.3)	—
Income status (monthly USD)	—	—	300 (220–350) [150–1,000]
Construction company	Buruuj	64 (33.5)	—
	Docol	46 (24.1)	—
	Al Bina	32 (16.8)	—
	Eameco	30 (15.7)	—
	Nawil	19 (9.9)	—
Job type	General labor	48 (25.1)	—
	Bricklayer & mason	33 (17.3)	—
	Armature fixing	30 (15.7)	—
	Concrete workers	24 (12.6)	—
	Painter	19 (9.9)	—
	Electrician	25 (13.1)	—
	Foreman	12 (6.3)	—
Years of work	<6 years	127 (66.5)	—
	≥6 years	64 (33.5)	—
Vibration exposure	Never	79 (41.4)	—
	Rarely (<1 h/day)	86 (45.0)	—
	Frequently (≥1 h/day)	26 (13.6)	—
Heavy lifting	Never	83 (43.5)	—
	Occasionally (<2 h/day)	52 (27.2)	—
	Frequently (≥2 h/day)	56 (29.3)	—
Bending	Rarely (<1 h/day)	49 (25.7)	—
	Occasionally (1–3 h/day)	91 (47.6)	—
	Frequently (>3 h/day)	51 (26.7)	—
Body mass index (kg/m^2^)	—	—	21.7 (15.4–33.8)
Smoking status	Non-smoker	128 (67.0)	—
	Smoker	63 (33.0)	—
Alcohol consumption	No	184 (96.3)	—
	Yes	7 (3.7)	—
Sleep pattern	≥6 h	134 (70.2)	—
	<6 h	57 (29.8)	—
Psychosocial distress	Low/moderate distress (≤5)	119 (62.3)	—
	High distress (>5)	72 (37.7)	—

### 3.2 Logistic regression analyses

From the univariable analyses, several variables were found to be significantly associated with low back pain (LBP) at *p* ≤ 0.20 (Table 3). These were heavy lifting, bending, body mass index, Years of work, sleep pattern, smoking status, income status, psychosocial distress, age, and vibration exposure. Results of the multivariable analysis are displayed in Table 4. In the final analysis, seven variables remained associated with LBP at p <0.05.

**Table 3:** Univariable analysis of associated factors with lower back pain (LBP) among construction workers in Mogadishu, Somalia, using a mixed-effects logistic regression model with construction company included as a random effect (*n* = 191).

Variable	Categories	Unadjusted OR	95% CI	*p*-value
Age category	18–28 years	1.00 (Ref)	—	<0.001
	29–40 years	5.11	2.39–10.90	
	≥41 years	4.95	1.91–12.81	
Education level	Any formal education	1.00 (Ref)	—	0.443
	No formal education	1.25	0.70–2.22	
Marital status	Unmarried	1.00 (Ref)	—	0.293
	Married	1.38	0.76–2.50	
Income status	Above poverty line	1.00 (Ref)	—	<0.001
	Below extreme poverty (<$3/day)	7.66	3.84–15.31	
Years of work	<6 years	1.00 (Ref)	—	<0.001
	≥6 years	6.62	3.23–13.54	
Body mass index (BMI)	Normal weight	1.00 (Ref)	—	<0.001
	Underweight	5.35	2.14–13.33	
	Overweight	3.19	1.36–7.49	
Smoking status	Non-user	1.00 (Ref)	—	<0.001
	User	7.53	3.58–15.87	
Alcohol consumption	No	1.00 (Ref)	—	0.709
	Yes	1.35	0.28–6.40	
Sleep duration	≥6 h	1.00 (Ref)	—	<0.001
	<6 h	4.34	2.15–8.73	
Heavy lifting Bending	Never	1.00 (Ref)	—	<0.001
	Occasionally (<2 h/day)	2.83	1.38–5.79	
	Frequently (≥2 h/day)	6.22	2.91–13.30	
	Rarely (<1 h/day)	1.00 (Ref)	—	<0.001
	Occasionally (1–3 h/day)	4.21	1.90–9.31	
	Frequently (>3 h/day)	10.10	4.02–25.36	
Vibration exposure	Never	1.00 (Ref)	—	0.012
	Rarely (<1 h/day)	1.74	0.93–3.28	
	Frequently (≥1 h/day)	4.06	1.49–11.05	
Psychosocial distress	Low/moderate distress (≤5)	1.00 (Ref)	—	<0.001
	High distress (>5)	4.93	2.58–9.45	

**Table 4:** Multivariable mixed-effects logistic regression of factors associated with LBP among construction workers in Mogadishu, Somalia, by using a mixed-effects logistic regression model with the variable construction company included as a random effect, *n* = 191.

Variable	Categories	Adjusted OR	95% CI	LRT *p*-value
Income status	Above poverty line	1.00 (Ref)	—	—
	Below extreme poverty (<$3/day)	7.60	2.48–23.28	<0.001
Years of work	<6 years	1.00 (Ref)	—	—
	≥6 years	4.85	1.56–15.11	0.006
Body mass index (BMI)	Normal weight	1.00 (Ref)	—	—
	Underweight	5.22	1.24–21.94	0.031
	Overweight	2.51	0.74–8.58	
Smoking status	Non-user	1.00 (Ref)	—	—
	User	5.70	1.94–16.78	0.002
Sleep duration	≥6 h	1.00 (Ref)	—	—
	<6 h	4.11	1.28–13.24	0.018
Heavy lifting	Never	1.00 (Ref)	—	—
	Occasionally (<2 h/day)	2.81	0.98–8.02	0.031
	Frequently (≥2 h/day)	4.29	1.26–14.56	
Bending	Rarely (<1 h/day)	1.00 (Ref)	—	—
	Occasionally (1–3 h/day)	2.50	0.78–8.02	0.046
	Frequently (>3 h/day)	5.35	1.33–21.45	

Results of the multivariable mixed-effects logistic regression analysis are presented in Table 4. Income status showed the strongest association with low back pain; workers living below poverty line (<$3/day) had over sevenfold higher odds of LBP (AOR = 7.60; 95% CI: 2.48–23.28) compared to those above the poverty line, though the wide confidence interval indicates statistical imprecision regarding the exact magnitude of effect. Similarly elevated odds were observed among workers with six or more years of construction experience (AOR = 4.85; 95% CI: 1.56–15.11) and smoking status (AOR = 5.70; 95% CI: 1.94–16.78) also markedly increased the odds of LBP.

Regarding ergonomic exposures, frequent bending (>3 h/day) and frequent heavy manual material handling were significantly associated with elevated odds of LBP (AOR = 5.35; 95% CI: 1.33–21.45 and AOR = 4.29; 95% CI: 1.26–14.56, respectively). Across these exposures, while lower confidence bounds consistently remained above unity, the wide intervals reflect the influence of sparse subgroup cells and sample size constraints. The intra-class correlation coefficient (ICC) was 2.8%, indicating that only a small proportion of variance in LBP was attributable to differences between construction companies.

## 4 Discussion

### 4.1 Prevalence of LBP

The 12-month period prevalence of low back pain among construction workers in Mogadishu estimated from the null mixed-effects model was 51.9% (95% CI: 43.5–60.3%). This estimate is comparable to prevalence rates reported among construction laborers globally, which range from 27.0 to 54.0% (10–12, 29). In particular, our findings closely mirror the figures observed in Nepal (52.0%) (9), Saudi Arabia (50.0%) (11), and Sweden (53.2%) (10). The persistently high burden of LBP across these diverse settings highlights the universally demanding nature of manual construction work, where excessive mechanical strain, heavy lifting, and awkward postures remain pervasive occupational hazards (1, 9, 10). Nevertheless, the lack of mechanization and extreme economic vulnerability characteristic of the Mogadishu construction sector may further compound these ergonomic risks by limiting recovery opportunities for local workers (9, 16). It should be noted, however, that this 51.9% period prevalence reflects workers active within medium-to-large registered construction companies. Musculoskeletal morbidity might manifest differently and potentially more severely among unregulated casual day laborers who lack continuous company attachment and formal job security.

### 4.2 Factors associated with LBP

Living below the extreme poverty line demonstrated the strongest association with low back pain in this study population (AOR = 7.60; 95% CI: 2.48–23.28). Although the wide confidence interval necessitates caution against interpreting the point estimate as an exact multiple of risk, the lower bound (2.48) indicates at least a two-to-threefold increase in adjusted odds. Workers enduring extreme economic precarity lack the bargaining capacity to decline hazardous, ergonomically strenuous tasks, which may expose them to continuous physical strain with limited nutritional and medical support. This finding is consistent with broader epidemiological literature demonstrating that low income and severe economic deprivation markedly elevate the risk of chronic back morbidity among manual laborers in developing settings (9, 30, 31).

In contrast, participant age was not independently associated with low back pain in the final multivariable model. This lack of association is likely explained by collinearity with cumulative work experience, as well as the “healthy worker survivor effect” frequently observed in heavy construction trades (32, 33). In physically demanding industries, older laborers who develop debilitating musculoskeletal pain often leave the workforce prematurely, leaving behind a healthier and more resilient cohort of older workers that masks the underlying relationship between chronological age and back pain (34, 35).

Underweight laborers exhibited higher adjusted odds of reporting low back pain compared to their normal-weight counterparts (AOR = 5.22; 95% CI: 1.24–21.94). While the interval around this estimate is broad due to the limited number of underweight participants in the sample, the finding aligns with the biomechanical hypothesis that insufficient lean muscle mass impairs spinal stabilization, transferring compressive forces directly to lumbar structures during heavy manual handling (36, 37, 59).

Tobacco use was also significantly associated with low back pain in this workforce (AOR = 5.70; 95% CI: 1.94–16.78). Nicotine exerts strong vasoconstrictive properties that can compromise microvascular circulation, reducing nutrient delivery and oxygen perfusion to lumbar tissues and intervertebral discs under continuous physical strain (38–41). Furthermore, our findings are consistent with primary observational investigations linking tobacco consumption to an increased occurrence of occupational back morbidity (42, 43).

Sleeping fewer than six hours per night was associated with an increased likelihood of reporting low back pain (AOR = 4.11; 95% CI: 1.28–13.24). Adequate rest is essential for physiological recovery following prolonged physical exertion, and chronic sleep deprivation has been shown to heighten pain sensitivity and impair musculoskeletal functional capacity. This observed association aligns with previous studies that have identified inadequate sleep duration as a key contributor to musculoskeletal complaints among manual laborers (9, 44–47).

Having six or more years of work in the construction sector significantly increased the odds of low back pain compared to fewer years of work (AOR = 4.85; 95% CI: 1.56–15.11). In unmechanized manual labor, prolonged duration of employment represents cumulative physical wear and continuous mechanical loading on the spine rather than protective technical proficiency. Over extended periods, chronic repetitive loading leads to micro-trauma and accelerated degenerative changes in spinal structures, consistent with occupational findings demonstrating an increased risk of back morbidity among long-term construction laborers (9, 48–50).

Heavy manual material handling was positively associated with low back pain in this workforce (AOR = 4.29; 95% CI: 1.26–14.56). Although statistical precision is constrained by sparse data across high-exposure categories, resulting in a wide confidence interval, the lower bound remains strictly above unity, indicating a notable increase in adjusted odds. Biomechanical and field investigations confirm that unassisted manual handling of heavy loads elevates intradiscal pressure and muscle fatigue, predisposing workers to lumbar strains and disc herniation (3, 37, 48, 51, 52).

Similarly, frequent trunk flexion exceeding 3 h per day was strongly associated with low back pain (AOR = 5.35; 95% CI: 1.33–21.45). While the broad confidence interval reflects sample size constraints in the highest exposure tier, the observed direction of effect consistently captures severe physical loading. Sustained trunk bending alters the natural lumbar lordosis, shifting support from stabilizing muscles to passive spinal ligaments and significantly increasing compressive loads on the intervertebral discs (2, 3, 10, 53, 54).

In contrast, whole-body vibration exposure was not retained in the final multivariable model, despite showing a significant association at the univariable stage (OR = 4.06; 95% CI: 1.49–11.05). In manual construction settings where mechanized equipment is rarely operated continuously, the potential influence of vibration is largely subsumed by the predominant and severe biomechanical demands of manual heavy lifting and awkward postures (22, 55–57).

Psychosocial distress was not significantly associated with low back pain after adjusting for other covariates in the final multivariable model. Although prospective cohort studies have demonstrated that perceived stress and workplace psychosocial stressors can independently contribute to low back disorders (33, 44), the lack of an independent statistical association in our study may reflect the overriding influence of severe ergonomic strain and extreme economic deprivation. In this unmechanized construction environment, the immediate physical strain resulting from frequent heavy manual handling and awkward trunk flexion likely represent predominant contributors to the occurrence of low back pain in this setting.

### 4.3 Study limitations

Several limitations are inherent in the present study and should be considered when interpreting the findings. First, the cross-sectional design restricts the ability to establish temporal cause-and-effect relationships, allowing only for the estimation of statistical associations between exposures and low back pain. Second, data collection relied on self-reports and interviewer-administered questionnaires (utilized to accommodate workers without formal education), which may have introduced measurement, interviewer, and recall biases. Social desirability could have led to an underreporting of sensitive behaviors such as tobacco and alcohol use during face-to-face interviews, whereas workers currently experiencing back symptoms may have systematically overreported the duration and intensity of their ergonomic exposures compared to their symptom-free peers.

Third, selection bias related to the healthy worker effect is possible. Because formal employee registries were unavailable, active daily attendance rosters served as the sampling frame, inevitably excluding laborers who were absent or had exited the workforce due to severe, debilitating musculoskeletal pain. Consequently, the estimated 51.9% prevalence may underestimate the true burden of low back pain across the broader construction workforce. Fourth, the study population comprised 191 manual laborers recruited across five major registered construction firms in urban Mogadishu. While focusing on established enterprises enabled rigorous roster-based probability sampling and achieved a high participation rate (95.0%), this recruitment strategy restricts the external validity of our findings. The occupational profile, job stability, and ergonomic exposures observed in these larger corporate entities may not fully represent workers engaged in smaller, unregistered building projects, informal residential worksites, or casual day laborers hired on a daily wage basis without company affiliation. Unregistered casual laborers frequently experience even more precarious economic conditions and less regulated physical work environments, which could alter musculoskeletal morbidity patterns. Furthermore, because data collection was geographically concentrated in the urban Banadir region, caution is warranted when generalizing these prevalence figures and effect estimates to construction laborers operating in rural or other emerging regions of Somalia. Finally, the modest analytical sample size of 191 participants inherently constrains statistical precision, as reflected in the wider confidence intervals observed for certain exposure subgroups in multivariable modeling. In addition, our investigation focused on individual-level ergonomic, lifestyle, and socio-demographic determinants, omitting organizational-level factors such as safety policy enforcement and workplace supervision. The omission of these organizational variables may have introduced residual confounding, potentially leading to an overestimation of the independent effects of individual-level ergonomic exposures. In light of these limitations, the findings of this cross-sectional study should be regarded as hypothesis-generating, and longitudinal designs such as prospective cohort studies are recommended to validate these associations. Lastly, although specifying Construction Company as a random effect accounted for workplace clustering, estimating variance components from only five clusters introduces imprecision into the random-effects parameters. Consequently, the intraclass correlation coefficient of 2.8% should be interpreted cautiously as an approximate estimate of between-company clustering.

## 5 Conclusion

This study demonstrates a high burden of low back pain among construction workers in Mogadishu, Somalia. Income status, body mass index, years of work, smoking status, sleep pattern, heavy lifting, and bending were identified as factors independently associated with low back pain in this workforce. Given the shared reliance on unmechanized manual labor and similar socioeconomic conditions across the region, these findings may reflect broader occupational health challenges in the Horn of Africa. To mitigate this burden, construction firms and industry stakeholders should prioritize practical, site-level ergonomic interventions—such as incorporating basic mechanical aids, implementing team-lifting protocols, and mandating scheduled rest breaks during continuous physical tasks. Finally, as this cross-sectional study is inherently hypothesis-generating, prospective longitudinal studies are recommended to validate these associations and evaluate the effectiveness of workplace interventions.

## Data Availability

The original contributions presented in the study are included in the article/supplementary material, further inquiries can be directed to the corresponding author/s.
